# Health, welfare and lifetime performance implications of alternative hatching and early life management systems for broiler chickens

**DOI:** 10.1371/journal.pone.0303351

**Published:** 2024-06-18

**Authors:** Hugo Hanna, Anne Richmond, Ursula Lavery, Niamh E. O’Connell

**Affiliations:** 1 Institute for Global Food Security, School of Biological Sciences, Queens University Belfast, Chlorine Gardens, Belfast, Northern Ireland, United Kingdom; 2 Moy Park Ltd, Portadown, Craigavon, Northern Ireland, United Kingdom; Tokat Gaziosmanpaşa University: Tokat Gaziosmanpasa Universitesi, TURKEY

## Abstract

Broiler chicks are typically hatched in a hatchery, exposing them to handling and transportation before being placed on the farm where (dry) feed and water is offered. This study compared different early life systems, including: (1) typical practice (control), (2) typical practice with wet feed offered upon placement, (3) access to water at the hatchery, (4) access to feed and water at the hatchery, (5) hatching on the farm. Birds were placed in groups of approximately 500 (day 0), with six replicates per treatment. Measures were taken between placement and slaughter (day 39) and included chick quality (navel and red hock scores), body weight, feed conversion ratio (FCR), mortality, gait and litter conditions scores, and behavioral and post-mortem assessments. There were no apparent treatment effects on gait score, play behaviour or novel object test measures, and no consistent effects on litter quality. Chick quality was only evaluated in Treatments 1 and 5 and was numerically worse in Treatment 5. Body weight at slaughter was lowest in Treatment 2, and did not differ between other treatments. Overall FCR was lowest (best) in Treatment 1, and did not differ between other treatments. There was higher overall mortality in Treatments 3 and 4 than in other treatments apart from Treatment 5. Treatment 4 appeared to promote feeding behaviour upon placement, and Treatment 5 birds rested the most, significantly more than in Treatment 2. Treatment 5 birds had the greatest bursa weights, and tibial dyschondroplasia appeared worse in Treatment 4. There were no consistent effects of early access to feed and water on gastrointestinal tract weight measures at slaughter. Compared to the control, there were few benefits in providing feed and/or water in the hatchery, or wet feed. Some benefits of in-house hatching were found, but negative effects were also apparent.

## Introduction

The standard commercial system for managing broiler chicks in Europe is to hatch eggs within a hatchery before grading, counting, vaccination and transport of the newly-hatched chicks to the broiler farm. Upon arrival at the farm the chicks are placed onto the floor, where for the first time feed and water is provided. However the chicks will still need to find both these essential resources to end the deprivation period. This approach enables the industry to tightly monitor the incubation and hatching environment, and to optimize labor and overhead costs. Chicks are thought to have a 72 hour supply of nutrients from their yolk sac, and thus do not require access to feed or water immediately after hatching [1, 2]. Under commercial conditions the window in which chicks hatch is 24–36 hours, and this is then followed by grading, vaccination, counting and transport of chicks, also taking several hours [3]. Therefore, some chicks which hatch early in the window may be over 2 days old before they are placed on the broiler farm.

Delaying access to feed and water by 24 to 48 hours, which is inherent with traditional hatching practices, has been suggested to be sub-optimal for bird health and performance [4–6]. It is thought to impair gastro-intestinal and organ development, and immune system functioning [6–8)]. Furthermore, delayed access to feed for 48 hours has been suggested to result in increased broiler mortality and lower growth rates compared to immediate access post-hatch [5]. It is also suggested that current commercial hatching systems can expose chicks to high levels of dust, pathogens and residual formaldehyde disinfectant during the hatching phase which can affect chick development [7], including relative bursal and spleen weights at day 0 [7]. Additionally, studies conducted within hatcheries demonstrated that darkness during incubation negatively affected the number of vocalizations, and immunoglobulin D and corticosteroid levels [9]. The hatchery handling, vaccination, sorting and counting process may also be a stressor to the chicks, increasing corticosteroids and fear-related behaviour, and affecting growth rates [10]. Finally, transportation has also been suggested to negatively affect body weight in the first 3 weeks of life and to negatively affect fear related behaviour [11, 12]. Therefore, the standard practices for hatching may have long-lasting negative impacts on the health, welfare and performance of broiler chicks.

In response to the potential flaws of the traditional early life management process, several variations to this system have been developed. One such system is designed to offer early access to feed, water and light to chicks upon hatching at a commercial hatchery [13, 14]. An alternative system exists whereby broiler hatching eggs are transported, on day 18/19 of incubation, to the broiler farm where they will hatch and birds will have immediate access to feed, water and light. Several variations of this system exist, whereby eggs are placed directly on the bedding, in baskets suspended above the litter, or in cardboard trays placed on the litter [15–20]. Furthermore, it has also been suggested that even in traditional hatching systems, providing chicks with a moist starter feed upon placement on the farm can improve early intestinal development and performance [21]. The alternative early life management systems described above are commercially available for implementation, however uptake within the industry, specifically the UK industry, appears low. There are a number of contributing factors including the level of capital investment required to alter existing systems, low levels of ‘new build’ hatcheries that could incorporate new approaches, and perhaps most importantly, a lack of information on effects of alternative systems on life time health, welfare, or performance.

The aim of this study was to evaluate the effects of different early life management systems on the life time health, welfare and performance of broiler chickens. The systems investigated involved providing different levels of access to resources at the hatchery (no feed or water, feed and water, just water), providing access to wet feed when chicks were placed on the farm after a traditional hatching process, or hatching birds on the farm. We hypothesized that reducing feed and water deprivation time in combination with the removal of hatchery processing and transport, in the case of in-house hatching, would result in improved health, welfare, and performance outcomes compared to all other treatments. We also hypothesized that early access to feed and water at the hatchery would have greater beneficial effects on outcome measures than access to wet feed on the farm, or access to water at the hatchery. Finally, we hypothesized that the control treatment would result in poorer outcomes than when feed and/or water was provided at the hatchery, or wet feed provided in-house.

## Materials & methods

This experiment was approved by the Faculty of Medicine, Health and Life Sciences (Queen’s University Belfast) Research Ethics Committee (reference number MHLS 20_117).

### Experimental design & housing

The study was carried out across a single production cycle between October and December 2020, under controlled conditions using a specifically designed, commercially-built broiler house in Northern Ireland. Broiler chicken eggs from the same batch produced by one 30 week old parent flock were randomly assigned to one of five treatments. Treatments 1–4, which were all hatched in a commercial hatchery, were as follows: (1) no access to feed or water at the hatchery (conventional commercial practice–‘Control’), (2) no access to feed or water at the hatchery but access to wet feed for 12 hours upon placement in the broiler house (‘Wet feed—Farm’), (3) access to water but no feed at the hatchery (‘Water—Hatchery’), (4) access to feed and water at the hatchery (‘Feed & Water–Hatchery’). Treatment 5 chicks were hatched at the broiler farm (‘In-house hatched’). Upon hatching or placement in the broiler house, all chicks had immediate access to water across all treatment groups, and to dry feed in ‘Control’, ‘Water—Hatchery’, ‘Feed & Water–Hatchery’, and ‘In-house hatched’ treatment groups. The ‘Wet feed—Farm’ chicks had access to wet feed for the first 12 hour and then dry feed from 12 hours post placement.

**Fig 1:**
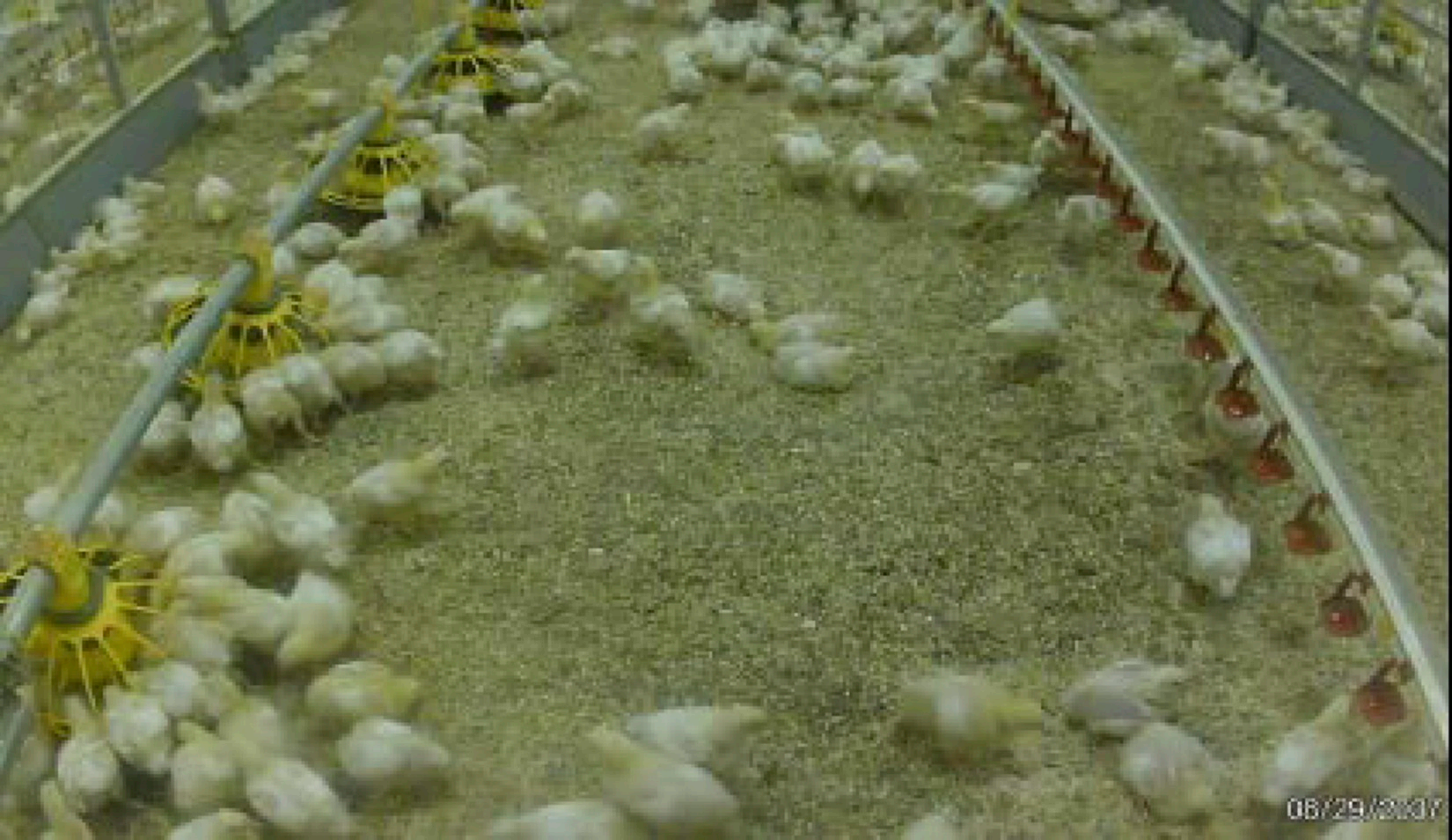
Feeder and drinker arrangement within the pens.

The broiler house was fitted with 36 (30 of which were used for this study) bespoke, purpose built pens divided into two rows of 18 split by a central passageway and with a further passageway around the perimeter of the house. All birds were reared simultaneously, sharing the same airspace and experiencing the same environmental conditions within the broiler house. The study consisted of six replicate groups per treatment. The treatment groups were evenly distributed across the house in a randomised block design. Each pen measured 3.56 x 6.88 m and was constructed from metal mesh gates with a plastic skirting board along the bottom. Each pen was set-up to mirror commercial conditions in terms of allocations of feeder and drinker space (Fig 1). All pens were fitted with Valco feeding systems and Valco FUZE ProLine pan feeders (Valco Industries, Inc, Pennsylvania, USA). Lubin 360 nipple drinkers (Lubing Maschinenfabrik GmbH & Co, Barnstorf, Germany) were fitted in all pens. Wood shavings were spread evenly across all pens as a litter material, to a depth of approximately 3–4 cm, prior to the study beginning. The ‘In-house hatched’ treatment litter was provided to a depth of 5 cm in areas where eggs were placed, providing added insulation to the egg, with litter depth in the rest of the pen remaining at 3–4 cm.

### Animal husbandry

The parent flock was located in Northern Ireland. Eggs required for Treatments 1–4 were transported to a commercial hatchery in England (which could facilitate the different hatchery treatments) while the eggs destined for the ‘In-house hatched’ treatment were held at a commercial hatchery in Northern Ireland, ensuring a simultaneous onset of egg incubation and an identical egg age across all treatment groups. Chicks in ‘Water—Hatchery’ and ‘Feed & Water–Hatchery’ treatments were offered water through drinking ‘gutters’ within the hatching basket, while feed was offered to ‘Feed & Water–Hatchery’ chicks via two feed troughs in the hatcher basket also (HatchTech, Innovatielaan 3, 6745 XW De Klomp, The Netherlands). These resources were available immediately from hatching in relevant treatments. Treatment 1–4 birds remained at the hatchery until the hatching process was complete. The time period from when the first chick hatched to placement of all hatchery-hatched chicks in-house was approximately 37 hours. The eggs assigned to the ‘In-house hatched’ group were incubated for 19 days at the hatchery. Infertile eggs were removed at both hatcheries. Eggs assigned to the ‘In-house hatched’ treatment were transported to the rearing house in a temperature controlled lorry. Upon arrival at the rearing house the eggs were placed by hand on top of the litter between the feeder and drinker lines of their respective pens. Upon hatching the ‘In-house hatched’ birds were then counted into boxes and graded using the same approach as at the hatchery. Chicks in Treatments 1–4 arrived at the rearing house from the hatchery on day 0. All birds were vaccinated for Infectious Bronchitis and Newcastle disease prior to placement/replacement. Birds were subsequently vaccinated for Gumboro through the water lines on day 17.

In total 15,108 Ross 308 broilers were used in this study. These were ‘as hatched’ and therefore a mix of males and females. A target bird number of 500 per pen was planned, providing a stocking density that did not exceed 38 kg/m^2^ at depopulation. The average number of birds in groups at the start of the rearing period was 509, 516, 489, 505 and 499 for Treatments 1–5, respectively. All pens associated with Treatments 1, 3, 4 and 5 contained feed placed on chick paper before either the eggs or the chicks were placed, as per commercial practice. Prior to the placement of ‘Wet feed—Farm’ chicks, the associated pens were set up with approximately half of their Day 0 feed allocation mixed with water (1 kg:1.5 liters) and placed on plastic trays. Approximately 12 hours post placement the plastic trays were removed from the pen with the remaining feed allocation offered as a dry feed, on chick paper. Except for this 12 hour period, the feed and water management was identical across the treatments during the rearing period. A commercial four stage feeding program was employed. Feed pans were topped up automatically and *ad libitum* access to water was provided. Both feeder and drinker heights were managed according to bird age and size.

A typical commercial lighting program was used with 1 hour of darkness provided on day 0, building up to a 6 hour block of darkness by day 7, which was maintained until 3 days prior to slaughter. The dark period was reduced to 3 hours, 3 days prior to slaughter, reducing by a further hour in each of the final 2 days pre-slaughter. The light intensity was managed according to commercial practice and maintained above the legal minimum of 20 LUX during the photophase. For the ‘In-house hatched’ treatment continuous lighting was provided from day 20 of egg incubation until the end of the hatch window on day 0. Prior to the ‘In-house hatched’ egg placement, the rearing house was pre-heated to approximately 36°C. Post egg placement the environmental temperature was driven by shell temperature, where a shell temperature of 35.5°C was achieved. From day 0 a typical commercial heating program was applied where the air temperature was incrementally reduced to 30°C on day 7. From day 7 the temperature was further reduced by 2°C for every 7 days until day 28. From day 28 the temperature was maintained at 24°C for the remainder of the rearing period.

In accordance with commercial practices, birds were thinned once on day 32 when they weighed approximately 1850 g. Upon thinning, 264 birds were removed from each pen while the remaining birds were slaughtered on day 39 at a body weight of approximately 2500 g.

### Measurements

#### Day old chick quality

On day 0 the chick quality of both the conventionally hatched ‘Control’ chicks and the ‘In-house hatched’ chicks was assessed. The scoring was conducted on the broiler farm for both treatments by the same observer as the chicks were being placed, or re-placed in the case of ‘In-house hatched’ chicks, into the pens. In total 150 chicks from each treatment, 25 from each group, were randomly selected. Hocks were assigned a score on a 1–3 scale where 1 = no red hocks, 2 = slightly red hocks, 3 = red hocks and skin possibly damaged [16]. Navels were also assigned a score on a scale of 1–3 [22], where 1 = clean and closed navel, 2 = black button less than 2mm or black string attached, and 3 = black button exceeding 2mm or open navel.

#### Bird performance

A total of 50 birds were randomly selected from each group and individually weighed on days 7 and 28 and an average body weight at each age determined. Upon thinning at day 32, 264 birds were removed from each pen and placed in a pen specific catching module. Each module was weighed providing an average body weight per bird. At clearance on day 39 this process was repeated with the remaining birds in each pen. The feed supplied to each pen was recorded on a weekly basis for the duration of the study. The broiler mortality and cull data were recorded on a daily basis from day 0 of production to final clearance and expressed as a percentage of the number of birds placed. Mortality was reported for the period up to 7 days, from day 8 to day 28, and across the whole experimental period. Pen level feed conversion ratio (FCR) was calculated using body weight and feed intake values for the following periods: 0–7 days, 0–28 days, and throughout the rearing period.

#### Gait score

On days 22, 29 and 36 twenty-five birds per group, totaling 150 per treatment, were selected at random and gently corralled using a portable gate. Each bird was then released sequentially and assessed by the same observer who assigned an individual gait score ranging from 1 (no defect) to 5 (complete lameness) using a modified Kestin method [23].

#### Behavioural measures

A novel object test was performed in each pen between 2 and 4pm on days 6, 20, 26 and 34. Different objects were used on each of the testing days as follows: an aluminium drinks can, a cardboard sweets box, a yellow sponge and a red rubber spoon. The novel object was placed at the center of each pen. The latency of the first bird to peck the object, the number of subsequent birds that made contact in the following 60 seconds, and the number of birds within 50 cm upon termination of the test was recorded. The test was terminated after 300 seconds and if no contact was made with the object during this time then the latency to contact was recorded as 300 seconds. The willingness to engage in food running play behaviour was also observed in every pen on days 6, 13, 20, 26 and 34 of production. The observer entered each pen and dropped a piece of paper straw of approximately 7 cm in length on to the litter before retreating to observe from outside the pen. The presence or absence of food running behaviour (as described in Table 1) with the straw was recorded.

**Table 1:** Ethogram used to classify general bird behaviour and play behaviour, based on Baxter et al. (2021) [24].

General Behaviour	
Foraging	Scratching and pecking at ground level from a standing position
Sitting Inactive	Sitting without performing any other behaviour with eyes open and head up. This includes lying on the chest or side.
Standing Inactive	Standing whilst not performing any other activity.
Resting	The bird is sitting with its head down on the ground or under its wing and exhibiting no other movement.
Feeding	The bird is engaged with the feeding pan with its head in the feeder.
**Comfort and Play Behaviour**	
Dustbathing	The bird is lying and performing head rubbing, vertical wing-shakes, leg scratching and/or raking the litter closer to it with its beak. Broilers clearly covered in litter and lying are categorised as dustbathing because the end of a dustbathing bout is typically signified by a body-shake, removing the litter. Broilers preening whilst covered in litter are recorded as dustbathing.
Food running	A bird follows and chases (runs at least two paces after another bird to begin the bout) a bird that has picked up or obtained a large object that projects from their beak. This bird has run from conspecifics but may make rapid and counter-intuitive direction changes towards conspecifics. There are conspicuous peeping noises that typically accompany this behaviour. The bout ends when the chasing bird loses interest and begins another behaviour e.g. sits down or begins feeding.
Running	A bird spontaneously runs, often including rapid changes of direction with no wing flapping involved. All birds exhibiting this behaviour within the defined area will be counted, regardless of where they start/stop. The behaviour will be classified as over when the bird returns to a walk or slower.
Frolicking	Spontaneous and rapid running and/or jumping and wing-flapping with no obvious intention, often with rapid direction changes. Running without wing-flapping is not classified as frolicking. A frolicking bout ends when the bird sits down or resumes another activity. Birds displaying frolicking directly leading to sparring are categorised as sparring, to avoid misinterpretation of their movements. All broilers exhibiting this behaviour within the frame will be counted, including those that started/finished outside of it.
Sparring	A bird simulates fighting behaviour with no obvious aggression or injurious contact. The following behaviours may begin a bout and occur during a bout: jumps with light kicking that make little or no contact with the receiver; stand-offs (threats) in which birds will face up to one another briefly, stepping close to one another and raising their necks to stand practically beak-to-beak (with or without a difference in head height); raising feathers around the neck, usually during a stand-off; stand-off with wing-flapping; stand-off with light pecks at the neck, head or beak of the receiving bird. These differ from aggressive actions in that they are not forceful and prolonged and they do not elicit strong avoidance from the receiver. It would be difficult to estimate a pecking order based on these behaviours. The bird that these behaviours are directed at may or may not respond, in some cases birds attempt a stand-off with a seated bird and are ignored. Birds usually end the short behaviour by sitting down or engaging in another activity.

The undisturbed behaviour of birds in each pen was recorded for 25 minutes by cameras (GeeKam, Shenzhen Bodalong Technology Co., China) mounted on tripods on days 1, 3, 13, 27 and 34. Recordings took place in five pens simultaneously (one from each treatment) before moving to the next five pens in a total of six recording bouts. Due to technical issues, one pen from the control treatment was not recorded on day 1. All recordings took place between 9.30am and 3pm and at a constant light intensity of 40 LUX. Focal scans were performed at 7 and 17 minutes of the recordings and used to count the birds in the selected area and to categorise their general behaviour according to the ethogram described in Table 1. Continuous observations of comfort and play behaviour were also performed over 2 minute periods beginning at 5, 10 and 15 minutes. During these observations the frequency of occurrence of sparring, food running, running, dustbathing or frolicking (described in Table 1) was recorded, and the total value observed for each behaviour over all three scans calculated. The floor area observed was consistent in all pens on each observation day and increased with bird age (for scan samples this equated to 3 m^2^ on days 1, 3, 13 and 27, and to 4.21 m^2^ on day 34; for continuous observations this equated to 3m^2^ on days 1, 3 and 13, to 4.21 m^2^ on day 27 and to 5.42 m^2^ on day 34).

### Litter quality

The litter quality was scored in every pen via visual inspection when birds were 16, 22, 29 and 36 days of age. The litter condition was classified on a scale from 1 to 5 as follows: (1) Dry and Friable, (2) Slightly moist (does not break apart), (3) Moist/Capped in small areas only, (4) Capped but now dry, (5) Capped and wet, and (6) Wet and soggy. Two scores were assigned to every pen on each of the observation days with the maximum litter score recorded used in analysis.

### Post-mortem assessments

Twenty birds from each treatment were randomly selected, immediately post-slaughter, upon thinning on day 32. At this stage it was possible to identify which treatment but not which pens the birds had belonged to. The birds were randomly selected across the duration of the slaughtering process for each treatment. All assessments were performed by the same veterinarian (blind to treatment) and this included determining the gender of the birds (‘Control’ 11 males/ 9 females, ‘Wet feed—Farm’ 10 males/10 females, ‘Feed & Water—Hatchery’ 10 males/10 females, ‘In-house hatched’ 14 males/6 females, ‘Water—Hatchery’ 7 males/13 females). The length of the gastrointestinal (GI) tract and the weight of the gizzard and GI tract, and of the heart, liver, pancreas, spleen and bursa were recorded. All organ weights were expressed as a percentage of the total body weight. Scores were also assigned for tibial dyschondroplasia (TD) and pododermatitis for each leg of all birds, with the highest score recorded. Pododermatites was scored on a scale where 0 = No Lesions, 1 = Mild Lesions and 2 = Severe Lesions [25]. The severity of TD was scored on a 4 point scale where 0 = normal growth plate, 1 = Mild lesions with cartilage development (<0.5 cm), 2 = Moderate lesion with abnormal cartilage development (0.5 cm-0.75 cm), and 3 = Severe lesion with cartilage (>0.75 cm) [26].

### Statistical analysis

All data were analysed using SPSS version 26 except for survival analysis which was performed using R v 4.0.1, and a significance level of P < 0.05 was used. A general linear model was used to assess treatment effects on the following variables (N = 30 for all): body weight at 7 and 28 days of age and at thinning, mortality between 7 and 28 days, total mortality, FCR at day 7 and total FCR. The number of birds originally placed in pens was used as a covariate in this analysis. Post hoc treatment comparisons were made using Fisher’s least significant difference test. Data on body weight at clearing (final slaughter) did not meet the assumptions of parametric statistics and was analysed using a Kruskal-Wallis test (denoted by H in the results section). A lack of homoscedasticity meant that a one way ANOVA with Welch’s correction was used to assess treatment effects on the following variables: 7 day mortality and 28 day FCR.

A linear mixed model (using restricted maximum likelihood analysis to produce final parameter estimates and post hoc comparisons) was used to assess main and interactive effects of treatment and age on a number of behavioural variables: the number of birds within 50 cm of the object at the end of the novel object test (N = 120), play and comfort behaviours recorded in continuous observations (N = 150), and all general behaviours recorded in scan observations (N = 149). In these analyses, ‘pen’ was included as a random factor, main effects were compared using a Bonferroni adjustment and an ante-dependence first order covariance structure was used. The number of birds originally placed in pens was used as a covariate in these analyses. Frolicking and running behaviours (continuous observations), and foraging, resting, feeding (scan observations) were square root transformed to improve normality of distribution prior to analysis, with back transformed means presented. Food running incidence during video continuous observations occurred too infrequently for statistical analysis.

Dustbathing and sparring behaviours (continuous observations) did not meet the assumptions for linear mixed model analysis following transformation, thus Kruskal-Wallis tests were used to assess treatment effects within different ages. There were insufficient data for statistical analysis of dustbathing on days 1, 3 and 13, and of sparring on days 1 and 3. No birds made contact with the novel object in the test at 6 days and very few made contact at 20 and 26 days (contact in 5 and 4 pens, respectively). Therefore statistical analysis of latency to contact the novel object and number of birds that made contact with the novel object within 60 seconds of first contact was not possible in these tests. The number of birds that made contact with the novel object within 60 seconds of first contact at 34 days could not be normalised and Kruskal-Wallis tests were used to assess treatment effects. The latency until birds made contact with the novel object on day 34 was analysed using a Cox proportional hazards regression using package Survival [27] in the R software package with right censoring at 300s, treatment as a fixed effect and number of birds placed in each pen as a covariate. Latency in the Control treatment was compared to latency in each of the other treatments. Assumptions of proportional hazards were met and the covariate met the assumption of linearity after a spline model was fitted. The birds engaged in food running behaviour when stimulated in the vast majority of tests (99.17%), and so there was insufficient variation in these data to perform statistical analysis.

Due to the ordinal nature of the data, treatment effects on chick quality scores (N = 300) were assessed using Mann Whitney U tests and within-week treatment effects on gait scores (N = 750 days 22 and 29, N = 749 day 36) and litter scores (N = 30) were assessed using Kruskal-Wallis tests. Treatment and sex effects on pododermatitis (N = 100) and tibial dyschondroplasia scores (N = 100) were also analysed using Kruskal-Wallis tests. The effects of treatment and sex on relative heart, gizzard, spleen and GI tract weight (N = 100) (calculated as a % of body weight) and GI tract length were determined using one way Analysis of Variance. Post hoc treatment comparisons were made using the Bonferronni test. Relative spleen and GI tract weight data were log transformed for analysis, and back transformed means are presented. The assumptions required for ANOVA were not met for analysis of treatment and sex effects on relative weight of the bursa, liver and pancreas (N = 100). In these cases treatment and gender effects were assessed using Kruskal-Wallis tests.

## Results

### Chick quality

There was no significant difference between the ‘Control’ and ‘In-house hatched’ treatments in hock score or in navel score, however there were numerical differences indicating poorer scores in the ‘In-house hatched’ treatment. The distribution of frequencies (%) was as follows: *Hock Scores*—‘Control’ Score 0 = 97.3, Score 1 = 2.0, Score 2 = 0.7; ‘In-house hatched’ Score 0 = 93.3, Score 1 = 5.3, Score 2 = 1.3; *Navel scores*–‘Control’ Score 0 = 82.0, Score 1 = 13.3, Score 2 = 4.7; ‘In-house hatched’ Score 0 = 72.0, Score 1 = 24.7, Score 2 = 3.3.

### Bird performance

Performance results are presented in Table 2. There was an effect of treatment on body weight at day 7 (F(4, 24) = 3.2, p = 0.030). ‘In-house hatched’ birds recorded the heaviest 7 day weight, significantly heavier than ‘Wet feed—Farm’ and ‘Water–Hatchery’ birds, while the 7 day weights of ‘Feed & Water–Hatchery’ birds were significantly heavier than ‘Wet feed–Farm’ birds. The 7 day weight of ‘Control’ birds did not differ from any other treatment groups. There was also a treatment effect on body weight at 28 days (F(4, 24) = 3.0, p = 0.038) where ‘Feed & Water–Hatchery’ and ‘In-house hatched’ birds were heavier than ‘Wet feed–Farm’ birds. The 28 day weight of ‘Control’ and ‘Water–Hatchery’ birds did not differ from any other treatments. The mean weight of birds on day 32 upon thinning did not differ between treatments. The average body weight of birds on day 39 at final clearing differed between treatments (H(4) = 10.1, p = 0.039). The lowest body weight at clearing was shown in the ‘Wet feed–Farm’ treatment which differed significantly from ‘Control’, Feed & Water–Hatchery’ and ‘In-house hatched’. No other groups differed from each other. Mean ranks (Median values in kilograms in brackets) were as follows: ‘Control’ = 21.17 (2.582), ‘Wet feed–Farm’ = 6.08 (2.499), ‘Water—Hatchery’ = 15.5 (2.549), ‘Feed & Water–Hatchery’ = 16.25 (2.562), ‘In-house hatched’ = 18.50 (2.575).

**Table 2:** Estimated marginal mean (±SE) values for different production measures, including bodyweight, mortality, and FCR.

	Treatment					p value
	Control	‘Wet feed—Farm’	‘Water—Hatchery’	‘Feed & Water—Hatchery’	‘In-house hatched’	
Body weight 7 days (kg)	0.165±0.002^abc^	0.162±0.002^a^	0.164±0.002^ab^	0.167±0.002^bc^	0.169±0.002^c^	0.030
Body weight 28 days (kg)	1.481±0.017^ab^	1.433±0.020^a^	1.498±0.020^ab^	1.522±0.017^b^	1.493±0.017^b^	0.038
Body weight 32 days (kg)	1.844±0.013	1.819±0.014	1.826±0.015	1.858±0.012	1.831±0.013	0.219
7 day mortality (%)*	0.36±0.092	0.38±0.111	0.70±0.161	0.76±0.114	0.87±0.224	0.076
8–28 day mortality (%)	0.49±0.187	0.49±0.210	1.16±0.219	0.92±0.182	0.56±0.185	0.134
Total mortality (%)	0.94±0.250^ab^	0.67±0.282^a^	2.31±0.294^c^	1.71±0.244^c^	1.62±0.249^bc^	0.011
7 Day FCR	0.763±0.008^bc^	0.726±0.008^a^	0.782±0.009^c^	0.774±0.007^bc^	0.759±0.007^b^	0.002
28 Day FCR*	1.301±0.012	1.323±0.012	1.301±0.027	1.291±0.011	1.295±0.010	0.418
Overall FCR	1.393±0.005^a^	1.411±0.006^b^	1.426±0.006^b^	1.419±0.005^b^	1.420±0.005^b^	0.003

*Analysed using one way ANOVA with Welch’s correction (mean values presented). General linear models used for all other analyses.

There were no significant treatment effects on the percentage of birds placed in pens that died between days 0 and 7, or between days 8 and 28. The percentage of birds that died across the entire experimental period differed between treatments (F(4, 24) = 4.1, p = 0.011), where the Wet feed–Farm’ treatment recorded lower mortality than all other treatments apart from ‘Control’. The ‘Control’ treatment had lower overall mortality than the ‘Water—Hatchery’ and ‘Feed & Water–Hatchery’ treatments, and no other significant treatment differences were found.

The FCR at 7 days differed significantly between treatments (F(4, 24) = 5.9, p = 0.002), with the ‘Wet feed–Farm’ treatment having a significantly lower FCR than all other treatments. The ‘Water–Hatchery’ treatment showed the highest 7 day FCR and this was significantly higher than that in the ‘In-house hatched’ treatment. The overall FCR was also affected by treatment (F(4, 24) = 5.4, p = 0.003), with ‘Control’ birds recording a significantly lower FCR compared to all other groups, which did not differ to each other. The FCR at 28 days did not differ significantly between treatments.

### Gait score

There was no significant difference between treatments in gait scores measured at day 22, day 29 or day 36. The distribution of birds in each gait score category at each age is available as supplementary material (S1 Table).

### Behavioural measures

#### Novel object test

There was no significant effect of treatment on the number of birds within 50 cm of the novel object at the end of the test, and no interaction between treatment and age. There was a significant effect of age (F(3, 99) = 33.4, p < 0.001), with the average number of birds within 50 cm of the novel object being higher at day 26 ((Estimated marginal means(EMM) ±SE) 17.3±0.86) than on all other days. The average number of birds within 50 cm of the object at the end of the test was also higher at 20 days (10.9±0.86) and at 34 days (9.2±0.86) than at 6 days (5.4±0.86). The number of birds that pecked the novel object in the 60 seconds following first contact did not differ between treatments at 34 days, and when compared with the control group, there was no difference in the latency for birds to make contact with the novel object at this age.

#### General behaviour (scan observations)

**Fig 2:**
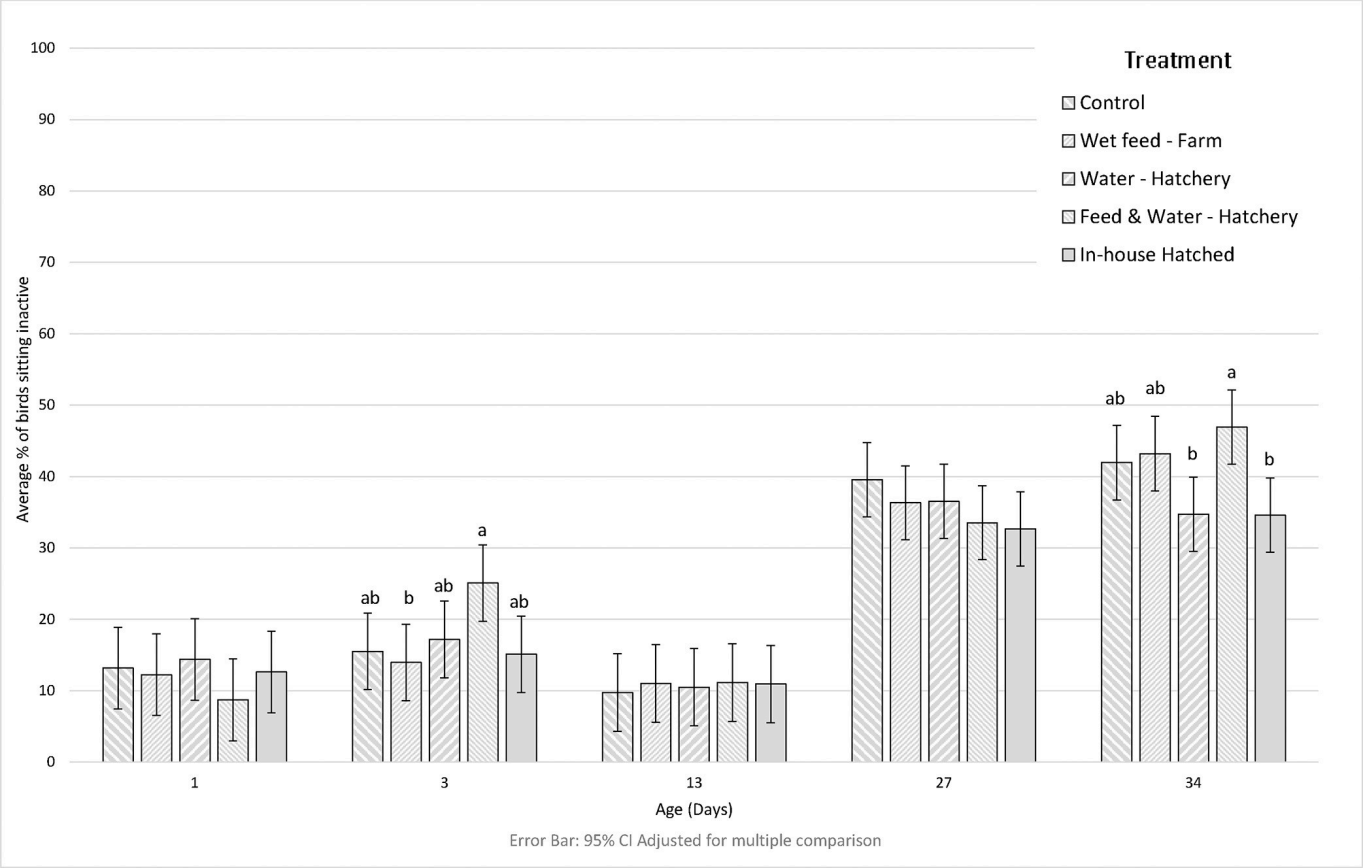
The mean proportion of birds sitting inactive in each treatment on each observation day.

**Fig 3:**
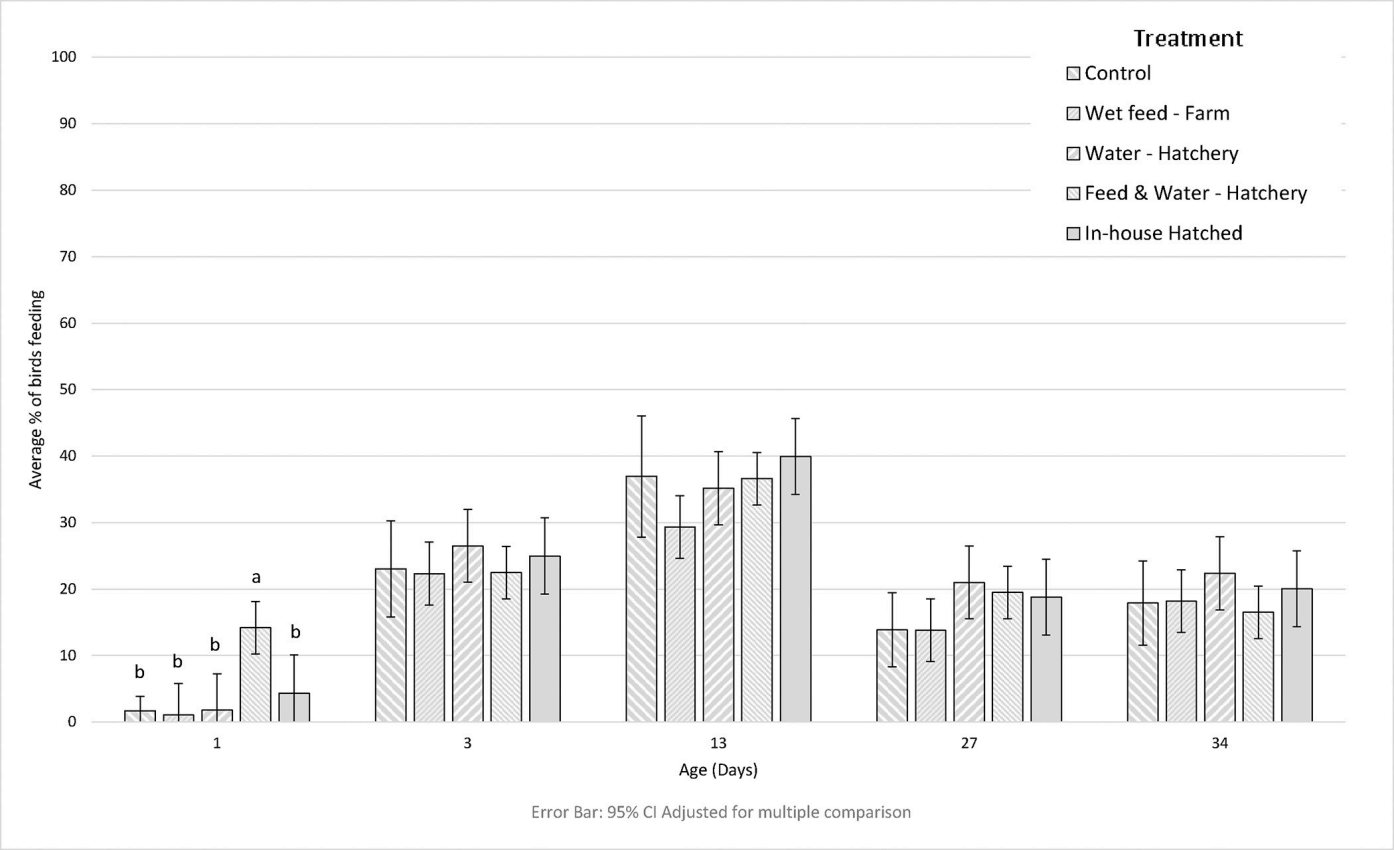
The mean proportion of birds performing feeding behaviour in each treatment on each observation day. Means and confidence intervals have been back transformed.

An overview of main effects of treatment and age, and of interactions, is provided in Table 3. Most resting was shown in the ‘In-house hatched’ treatment, and this was significantly greater than in the ‘Wet Feed–Farm’ treatment (F(4, 123) = 3.0, p = 0.021). There were interactions between treatment and age in sitting inactive (F(16, 123) = 1.9, p = 0.030) and feeding behaviour (F(16, 123) = 2.0, p = 0.021) (Table 3). On day 3 a higher proportion of ‘Feed & Water–Hatchery’ birds were observed sitting inactive compared to ‘Wet feed—Farm’ birds, with no further differences observed (Fig 2). The sitting inactive behaviour of ‘Feed & Water–Hatchery’ birds peaked again on day 34, significantly higher than ‘In-house hatched’ and ‘Water—Hatchery’ birds (Fig 2). On day 1 a greater proportion of ‘Feed & Water—Hatchery’ birds were observed performing feeding behaviour than in all other groups (Fig 3).

**Table 3:** Estimated marginal means (±95% CI) for the % birds performing general and play behaviours by treatment and by observation day.

Behaviour	Treatment					Age					P value		
	Control	Wet feed- Farm	Water—Hatchery	Feed & Water—Hatchery	In-house hatched	1	3	13	27	34	Treat.	Age	Treat. x Age
General behaviour													
Sitting inactive	23.99 (21.56, 26.42)	23.36 (20.67, 26.04)	22.66 (19.87, 25.46)	25.09 (22.77, 27.41)	21.19 (18.83, 23.56)	12.24^a^ (9.87, 14.60)	17.38^b^ (15.06, 19.70)	10.68^a^ (8.36, 13.00)	35.72^c^ (33.40, 38.04)	40.28^c^ (37.96, 42.60)	0.230	<0.001	0.030
Standing inactive	5.47 (4.10, 6.84)	5.73 (4.22, 7.25)	4.54 (2.96, 6.12)	5.88 (4.57, 7.20)	4.00 (2.67, 5.34)	9.04^c^ (7.71, 10.38)	3.27^a^ (1.96, 4.58)	7.06^bc^ (5.75, 8.37)	4.39^ab^ (3.08, 5.70)	1.86^a^ (0.55, 3.17)	0.331	<0.001	0.943
Foraging*	5.72 (4.00, 7.76)	8.16 (5.87, 10.84)	5.63 (3.68, 7.98)	4.13 (2.74, 5.79)	5.53 (3.87, 7.47)	4.48^a^ (3.31, 6.67)	4.73^a^ (3.23, 6.50)	10.07^b^ (7.82, 12.59)	5.87^ab^ (4.19, 7.82)	4.14^a^ (2.75, 5.81)	0.079	<0.001	0.824
Resting*	8.63^ab^ (6.42, 11.17)	5.99^a^ (4.00, 8.37)	11.14^ab^ (8.25, 14.47)	8.87^ab^ (6.72, 11.32)	12.46^b^ (9.84, 15.39)	31.16^e^ (26.92, 35.57)	22.77^d^ (19.24, 26.60)	0.18^a^ (0.00, 0.65)	2.87^b^ (1.71, 4.33)	7.63^c^ (5.65, 9.91)	0.021	<0.001	0.069
Feeding*	16.21^ab^ (13.52, 19.15)	14.67^a^ (11.85, 17.78)	18.87^ab^ (15.54, 22.53)	21.22^b^ (18.26, 24.41)	19.73^ab^ (16.84, 22.87)	3.62^a^ (2.44, 5.03)	23.83^c^ (20.69, 27.21)	35.51^d^ (31.64, 39.59)	17.26^b^ (14.60, 20.14)	18.94^bc^ (16.15, 21.96)	0.027	<0.001	0.021
Play behaviour													
Frolicking*	12.80 (9.84, 16.27)	12.55 (9.29, 16.31)	9.72 (6.76, 13.21)	14.81 (11.69, 18.29)	12.50 (9.61, 15.78)	12.61^b^ (9.70, 15.90)	13.19^b^ (10.26, 16.48)	32.64^c^ (27.93, 37.72)	6.96^a^ (4.88, 9.41)	4.39^a^ (2.78, 6.37)	0.322	<0.001	0.785
Running*	14.22 (10.89, 18.00)	16.66 (12.68, 21.19)	15.22 (11.28, 19.75)	17.46 (13.89, 21.43)	17.39 (13.78, 21.43)	26.39^b^ (21.89, 31.32)	26.66^b^ (22.21, 31.51)	35.92^b^ (30.72, 41.51)	4.97^a^ (3.17, 7.18)	2.50^a^ (1.28, 4.13)	0.662	<0.001	0.848

CI, Confidence intervals adjusted for multiple comparison*Back transformed means and confidence intervals presented

Where there were no interactions, main effects of age were shown in standing inactive behaviour (F(4, 123) = 18.9, p < 0.001) and foraging behaviour (F(4, 123) = 5.7, p < 0.001), but no consistent age-related patterns were apparent.

### Play and comfort behaviour (continuous observations)

No main effect of treatment or interaction effects were observed for frolicking or running. There were significant effects of age on frolicking (F(4, 123) = 40.7, p < 0.001) and running (F(4, 123) = 75.3, p < 0.001), with both behaviours generally declining towards the end of the production cycle (Table 3). Within each age there were no significant treatment effects on sparring or on dustbathing.

### Litter quality

There was no significant difference between treatments in the maximum litter score recorded on day 16, day 22 or day 36. There was an effect of treatment on litter scores on day 29 where both the ‘Control’ and ‘Wet feed–Farm’ treatments differed significantly from the ‘In-house hatched’ and ‘Water—Hatchery’ groups, but no other significant treatment effects were shown (Mean ranks: ‘Control’ 22.42, ‘Wet feed–Farm’ 20.08, ‘Water–Hatchery’ 10.58, ‘Feed & Water–Hatchery’ 13.83, ‘In-house hatched’ 10.58, H(4) = 11.4, p = 0.023). For ease of interpretation, average maximum scores for litter quality on day 29 were as follows: ‘Control’ = 5.3, ‘Wet feed–Farm’ = 5.0, ‘Water–Hatchery’ = 4.17, ‘Feed & Water–Hatchery’ = 4.5, ‘In-house hatched’ = 4.17.

### Post-mortem assessments

Pododermatitis score at thinning was not significantly affected by treatment (p = 0.063), although ‘Feed & Water–Hatchery’ and ‘In-house hatched’ treatments had fewer birds in the highest score category. Pododermatitis was not affected by gender. The distribution of frequencies (%) for pododermatitis scores in different treatments was as follows:—‘Control’ Score 0 = 5, Score 1 = 70, Score 2 = 25; ‘Wet feed—Farm’ Score 0 = 30, Score 1 = 35, Score 2 = 15; ‘Water—Hatchery’ Score 0 = 10, Score 1 = 70, Score 2 = 20; ‘Feed & Water—Hatchery’ Score 0 = 15 Score 1 = 85, Score 2 = 0; ‘In-house hatched’ Score 0 = 20, Score 1 = 80, Score 2 = 0. Tibial dyschondroplasia (TD) scores were significantly influenced by treatment (H(4) 15.7, p = 0.003), however not by gender (p = 0.570). The ‘Feed & Water—Hatchery’ birds were found to have a significantly worse TD score compared to all other treatments (p < 0.05) (Mean ranks: ‘Control’ 45.95, ‘Wet feed–Farm’ 45.95, ‘Feed & Water–Hatchery’ 63.80, ‘In-house hatched’ 50.85 ‘Water–Hatchery’ 45.95). The distribution of frequencies (%) for TD scores were as follows:—‘Control’ Score 0 = 95, Score 1 = 5, Score 2 = 0, Score 3 = 0; ‘Wet feed—Farm’ Score 0 = 95, Score 1 = 5, Score 2 = 0, Score 3 = 0; ‘Feed & Water—Hatchery’ Score 0 = 60, Score 1 = 30, Score 2 = 10, Score 3 = 0; ‘In-house hatched’ Score 0 = 85, Score 1 = 15, Score 2 = 0, Score 3 = 0; ‘Water—Hatchery’ Score 0 = 95, Score 1 = 5, Score 2 = 0, Score 3 = 0.

Relative bursa weight was affected by treatment (H(4) = 32.8, p < 0.001), and was greater in the ‘In-house hatched’ treatment than all other treatments. Birds in the ‘Control’ and ‘Wet feed–Farm’ treatments had greater relative bursa weights compared to those in ‘Feed & Water—Hatchery’ and ‘Water—Hatchery’ treatments, while ‘Water—Hatchery’ birds did not differ from ‘Feed & Water—Hatchery’ birds, and ‘Control’ and ‘Wet feed–Farm’ treatments did not differ (Mean ranks (Median (%)): ‘Control’ 56.80 (0.123), ‘Wet feed–Farm’ 53.25 (0.116), ‘Water–Hatchery’ 34.85 (0.086), ‘Feed & Water–Hatchery’ 30.70 (0.074), ‘In-house hatched’ 76.90 (0.167)). Relative liver weight was affected by treatment (Mean ranks (Median (%)): ‘Control’ 69.60 (3.895), ‘Wet feed–Farm’ 56.50 (3.788), ‘Water–Hatchery’ 53.90 (3.745), ‘Feed & Water–Hatchery’ 32.93 (3.365), ‘In-house hatched’ 39.58 (3.389)), (H(4) = 20.0, p < 0.001) and was greater in ‘Control’ birds than in ‘Feed & Water—Hatchery’ and ‘In-house hatched’ birds, while ‘Water–Hatchery’ and ‘Wet feed-Farm’ displayed heavier relative liver weights compared to ‘Feed & Water–Hatchery’ birds. Treatment also affected relative GI tract weight (F(4, 95) = 6.4, p < 0.001), where ‘Control’ and ‘Feed & Water—Hatchery’ birds had greater relative weights compared to ‘Water–Hatchery’ and ‘In-house hatched’ birds, while ‘Wet feed–Farm’ didn’t differ to any other treatment (Mean % (±95% CI): ‘Control’ 4.68 (4.40, 4.96), ‘Wet feed–Farm’ 4.28 (4.04, 4.54), ‘Water–Hatchery’ 4.09 (3.87, 4.33), ‘Feed & Water–Hatchery’ 4.73 (4.46, 5.02), ‘In-house hatched’ 4.13 (3.94, 4.33)). There was no significant effect of treatment on remaining measures. The relative Gizzard weight (F(1, 98) = 7.5, p < 0.01) and GI tract length (F(1, 97) = 8.2, p < 0.01) were affected by gender (Mean±SE male/female: relative gizzard weights (%) 1.625±0.024 and 1.733±0.033; GI tract length (cm) 227.21±2.584 and 215.12±3.452, respectively). No other significant gender effects were observed.

## Discussion

The purpose of this study was to assess the effects of various early life management systems on broiler health, welfare, and performance. Previous studies suggest that a lack of handling and transport after hatching might lead to improved growth in broilers, particularly in the early phase of rearing [11, 28]. A comparison of body weight data between the ‘Feed & Water—Hatchery’ and ‘In-house hatched’ treatments in the current study suggest that these effects were limited. Holleman’s et al. (2018) also did not find any significant difference in body weight as a result of transport [12]. Although there were numerical differences, our results indicate no significant detrimental effect of delayed access to food and water on body weight at 7 days. It is possible that any effects on body weight of early access to feed and water may have dissipated somewhat by this point, as some studies suggested improved early growth may not persist [15, 16]. Indeed while Jessen et al. (2021) found a significant difference in growth in the first 48hrs between in-house hatched and traditionally hatched birds, by day 7 the difference between the treatments was 3 g in favour of in-house hatched [29]. This study reported a very similar effect, indicating a 4 g benefit in favour of the ‘In-house hatched’ treatment. The lowest 7 day weight was with the ‘Wet feed—Farm’ treatment and this may have been due to the fact that mixing water with the starter crumb diluted the nutritional density of the mixture. This effect appeared to persist and at day 39 birds in the ‘Wet Starter—Farm’ treatment were significantly lighter, a finding somewhat similar to a previous study with broilers, whereby a water/feed combination offered at the hatchery produced a numerically lower final weight compared to traditionally managed birds with no feed/water at the hatchery [30].

The current study found that the FCR at 7 days was significantly lower in ‘Wet Feed–Farm’ birds compared to all other groups. Kidd et al. (2007) found that a feed/water combination provided at the hatchery resulted in a numerically lower 17 day FCR compared to traditionally hatched birds, somewhat in agreement with this study’s findings [30]. It is unclear why the current study found the 7 day FCR of the ‘Water- Hatchery’ birds to be significantly poorer than some other treatments, particularly when the limited work to date suggests the effect of early access to water on performance is highly transient [5]. In terms of overall FCR, the ‘Control’ treatment had a significantly lower value than all other treatment groups, and our results suggest that the ‘Control’ group began to deviate from the other groups from 28 days onwards. Based on these findings it can be suggested that an earlier onset development of the GI tract and organs as a result of earlier access to feed and water did not positively influence final FCR. This corresponds with research by Özlü et al. (2020) and Kang et al. (2018) who found numerically lower overall FCR for birds with delayed access to feed and water post hatch (40–48 hours) [31, 32]. Neither study reflected on the reason for this finding, therefore further work is required to better understand these findings, and why the same effect was not observed in the ‘Wet Starter—Farm’ treatment group in the current study.

Previous studies have found significantly poorer navel and hock scores with in-house hatching compared to traditional hatching [15, 16, 28, 33]. The incidence of red hocks and/or poorly healed navels is widely regarded to be caused by sub-optimal incubation regarding temperature and relative humidity [34–38]. This can cause prolonged pushing of the hocks against the shell wall during hatch and poorly healed navels with the yolk sac drying too quickly, causing a string to protrude from the navel [34, 36–38]. The findings reported earlier [15–17, 28, 33] were observed using systems where the egg was held above the litter, either in cardboard egg trays or in plastic hatchery egg trays, whereas our results were derived from a system where the eggs were placed on, and partially surrounded by the litter. It is possible that the residual heat in the litter and concrete floor acted as a buffer to more pronounced fluctuations of air temperature in the current study, leading to a lack of significant treatment differences. Poor navel and hock scores have serious implications for bird welfare as both are indications of thermal stress experienced during incubation [34, 38]. While no significant differences or statistical tendencies were shown in the current study, scores were numerically higher in the in-house hatched treatment, and this, together with previous research, suggest that in-house hatching is a risk factor for poorer chick quality and that management of temperature and humidity in these systems will be key to mitigating this risk.

The mortality findings mirrored the chick quality findings where no significant differences between traditional hatching and in-house hatching were found on early or overall mortality. Some studies have reported reduced mortality with in-house hatching compared to traditional hatching in the first week of life and over the rearing period [16, 33]. However, de Jong et al. (2019) Molenarr et al. (2023) and Souza da Silva et al. (2021) found no significant difference in mortality between hatchery hatching and in-house hatching [15, 28, 39], concurring with this study’s findings. It is worth indicating, however, that we only recorded mortality data from when the birds were placed on the broiler farm. Souza de Silva et al. (2021) found first week and total mortality was numerically higher with birds offered feed and water in the hatchery compared to traditional hatching [28]. This is similar to the current study’s findings where ‘Control’ birds suffered significantly lower total mortality than ‘Food & Water–Hatchery’ birds. This suggests that the combination of early feed and water access and transport may be less favorable, increasing stress, and may be a factor contributing to higher mortality [28]. Interestingly, the fact that significant differences between our treatments were only shown across the entire production cycle (and not specifically in the first week) suggest that these effects persist. The findings from this study suggest that these effects also occur when just water is provided in the hatchery. This is in contrast to previous suggestions that offering water (only) in the hatchery provided a transient benefit in terms of mortality [5]. Further research into the causes of these mortalities is warranted, but overall our results do not show benefits of post-hatch access to feed and/or water in reducing mortality.

A treatment effect was observed on tibial dyschondroplasia (TD), where the ‘Feed & Water—Hatchery’ group displayed a worse TD score compared to all other treatment groups. The incidence of TD is linked with rapid growth rates [40, 41], which may partly explain our findings as ‘Feed & Water—Hatchery’ birds were, numerically, amongst the heaviest on day 7 and the heaviest on days 28 and 32. The results suggest that the promotion of rapid growth in the early and mid-stages of production may compromise leg health. The difference in TD score was not reflected in any treatment differences in gait score, however. This is consistent with the literature whereby de Jong et al. (2019; 2020) and Jessen et al. (2021) observed no difference in gait score between in-house and traditional hatching in both a commercial and a research facility setting, while Giersberg et al. (2021) also found no influence of hatching system (In-house hatching or feed and water at the hatchery) on gait score [15–17, 33]. The recent literature on the effect of hatching system on subsequent pododermatitis is somewhat varied, with some studies reporting reduced pododermatitis with in-house hatching and in-hatcher access to feed and water [15, 17]. Other studies comparing traditional hatching and in-house hatching reported similar findings to this study, indicating a tendency for improved pododermatitis scores with in-house hatching but no significant effects [16]. Because broiler chickens spend the vast majority of their lives in contact with the litter, the litter quality has a significant effect on bird health, including foot health, where excessive litter moisture increases pododermatitis [42]. The lack of treatment effect on pododermatitis in this study may reflect the fact that litter quality did not differ significantly between treatments except at one time period. Previous findings on the effect of hatching system on litter quality are also varied. Some studies have found improved litter score with in-house hatching when compared to traditional hatching [15], whereas others indicated no effect of system on litter quality under more controlled experimental conditions [16, 17].

Previous studies have reported conflicting results regarding fearfulness of broilers and early life system. For example, Giersberg et al. (2021) reported no difference between systems, while Giersberg et al. (2020) reported hatchery-hatched birds were less fearful than in-house hatched birds [3, 17]. In contrast, Jessen et al. (2021) reported that in-house hatched birds were less fearful compared to hatchery-hatched and Hedlund (2019) concurred, reporting hatchery processed and transported chicks were more fearful than those that weren’t [10, 29]. There are different tests for determining fearfulness in broilers, including exposure to a novel environment and/or novel object, and tonic immobility and human approach tests, amongst others. The previously mentioned literature used a combination of these tests to assess fearfulness, partly explaining the differing results. However when we focus solely on the novel object test as was used in our study, Jessen et al. (2021) found no significant difference between in-house hatched and traditionally hatched birds, and Giersberg et al. (2021) found no difference between hatchery-hatched, hatchery-hatched with food available upon hatching, and in-house hatched birds [17, 29]. Only Giersberg et al. (2020), using a novel object test, found traditionally hatched birds were less fearful than in-house hatched birds [3]. While the current study is not in agreement with all of the literature, it does concur with the majority of findings, suggesting no significant effect of hatching system on novel object test behaviour. Unfortunately in this study the broilers did not always engage with the test, and a novel object test does not differentiate between fearfulness and indifference, therefore other experimental approaches may have been more appropriate.

There is very little scientific information on early life management and its consequence on play behaviour in broiler chickens, however our study showed no treatment effects. Additionally, the proportion of birds observed foraging/floor pecking was found to be not significantly affected by treatment, supporting findings by Giersberg et al. (2020), who compared in-house hatching to traditional hatching [3]. A further study found that increasing post hatch food and water deprivation increased levels of activity and decreased levels of sitting/standing inactive behaviour [43]. Our findings did not fully reflect this, although ‘Feed & Water–Hatchery’ birds were observed performing more sitting inactive behaviour on day 3 compared to ‘wet feed–Farm’ birds, and on day 34 when compared to ‘Water–Hatchery’ and ‘In-house hatched’ groups.

This study found that the greatest level of resting behaviour was shown by the ‘In-house hatched’ chickens, significantly greater than the ‘Wet feed–Farm treatment. Although there was no significant interaction, this effect was particularly evident on day 1 (see S1 Fig). Jessen et al. (2021) also found comparable results suggesting that in-house hatching provides a conducive environment for resting post-hatch as the birds suffer few potential stress events [29]. It is possible that ‘In-house hatched’ chicks may be more at ease with their environment, having stayed where they hatched and already found food and water sources, potentially promoting a greater level of resting behaviour. A further factor to be considered, however, is the environment that hatchery-hatched chicks experience. During the final stages of incubation within a hatchery and during transport chicks are typically kept in darkness with limited room for movement, therefore it may not be surprising these birds tend not to rest when stimulated by light, space and the introduction to a more complex environment. As such, the resting behaviour differences between treatments may be less to do with in-house hatching promoting rest and more to do with the traditional system stimulating activity. A further factor to consider is the lighting programme that in-house hatching birds experienced. During the final stages of incubation and throughout the hatching process, in both the current study and that by Jessen et al. (2021), the birds were under continuous lighting [29]. It is therefore possible that disturbance caused by the prolonged photoperiod (day 18 of incubation to day 3 post hatch) may have resulted in increased resting behaviour under in-house hatching conditions. It may be reasonable to suggest, therefore, that the cause of the observed increase in resting and its relationship with bird welfare warrants further investigation.

Our results also indicate a treatment effect in the proportion of birds eating, where the ‘Feed & Water—Hatchery’ birds performed more feeding behaviour on day 1. The introduction of feed within the hatchery in this treatment would have initiated GI tract and organ development, and the potential lack of feed intake during transport due to stress or darkness may have stimulated feeding behaviour when the chicks were placed. A recent study found that in-house hatched chicks displayed significantly more feeding behaviour [29], however this was not supported by this or other research [3].

The ‘In-house hatched’ group displayed a significantly heavier bursa compared to all other treatment groups. This suggests reduced stress and improved immune status [44, 45] and although this did not translate into the lowest mortality figures in the current study, it could have important implications for birds under greater disease pressure. Previous work suggests that early feeding promotes a significantly heavier bursa [8, 46, 47]. This is a result of early feeding promoting early development of the GI tract, and the gut-associated lymphoid tissue and organs [8]. This does not explain, however, why a heavier bursa was not also found in the ‘Feed & Water–Hatchery’ treatment in our study. There may therefore be other reasons for this treatment effect on bursal weight. It is possible that the fact that we could not randomise our samples across pens affected this and other post mortem results, however we sampled birds across the slaughter period to try to ensure that different pens were represented. Further research on the effects of in-house hatching on bursal weight and immune status is clearly warranted. A recent meta-analysis suggests that organ and intestinal weights are negatively impacted by feed and water deprivation during the first week of life [5], however the effect was transient with only a limited number of studies reporting long term effects. Our study did not show significant increases in organ weight at thinning when ‘Feed & water–Hatchery’ birds were compared with the ‘Control’ and ‘Wet feed–Farm’ birds. In fact in some cases (for example with liver and bursa weight) values were significantly increased for the ‘Control’ and ‘Wet feed–Farm’ birds treatments. The reason for this is not clear. Van de Ven et al. (2011) noted that hatch timing influenced organ weights until at least 21 days of production [48]. It is possible that different hatch timings influenced organ weights to a greater extent than early access to food and water in the current study, though it is unlikely as birds were hatched in the same environment and chicks from across the hatching window were allocated to each hatchery-hatched treatment. It is also possible that our findings did not concur with previous literature as a consequence of age at sampling. Dibner et al. (1998) observed a positive effect of early feeding on broiler chicken bursal and spleen development at 21 days [46]. This finding is in agreement with Simon et al. (2014), who found that broilers provided with early access to food had a numerically larger bursa on day 21 compared to birds with delayed access to food, however by day 35 and 42 the effect had reversed [49].

## Conclusion

This study demonstrated that in-house hatching did not generally improve the overall performance of broilers in terms of liveweight or mortality compared to most other treatments, and resulted in poorer FCR than the control. The provision of feed and water, or water only, at the hatchery did not affect liveweight but had an adverse effect on mortality and FCR relative to traditional hatching. In terms of health, although numerically poorer, in-house hatching did not significantly compromise chick quality, and these birds showed increased bursa weights compared to all other treatments. Welfare indicators offered a mixed view regarding the early fed treatments (‘In-house hatched’ and ‘Feed & Water–Hatchery’) with both showing a tendency for lower pododermatitis scores, however the ‘Feed & Water–Hatchery’ birds also displayed a significantly higher prevalence of TD compared to birds in other treatments. Behavioural differences were subtle, however the ‘Feed & Water—Hatchery’ birds displayed more feeding, while more ‘In-house hatched’ birds were resting on day 1. The welfare implications regarding resting remain unclear, warranting further investigation. Offering wet feed at the farm was associated with a poorer final weight and overall FCR. This study highlighted that alternative early life management systems significantly impact lifetime health, welfare and performance. In-hatchery access to feed and water, or water only, or access to wet feed upon placement on the farm were associated with negative effects on several measures compared to the control treatment. It is apparent from this study that the findings related to in-house hatching were inconsistent, with both positive and negative outcomes compared to traditional hatching.

## Supporting information

S1 Fig Average proportion of birds resting. (DOCX)

S1 Table Distribution of frequencies of gait scores. (DOCX)
