# Supplementary material for: Health, welfare and lifetime performance implications of alternative hatching and early life management systems for broiler chickens
Source: PLoS One. 2024 Jun 18;19(6):e0303351. doi: 10.1371/journal.pone.0303351 (PMC11185489; doi:10.1371/journal.pone.0303351)
Supplement: S1 Fig — (DOCX) [file pone.0303351.s001.docx]

Error Bar: 95% CI Adjusted for multiple comparison

Treatment

**Supplementary Figure 1.**
