# Supplementary material for: Health, welfare and lifetime performance implications of alternative hatching and early life management systems for broiler chickens
Source: PLoS One. 2024 Jun 18;19(6):e0303351. doi: 10.1371/journal.pone.0303351 (PMC11185489; doi:10.1371/journal.pone.0303351)
Supplement: S1 Table — (DOCX) [file pone.0303351.s002.docx]

**Supplementary Table 1**

**The average distribution of birds (%) from each treatment in each gait score category**

| Treatment | Day 22 |  |  |  |  |  |
| --- | --- | --- | --- | --- | --- | --- |
|  | GS0 | GS1 | GS2 | GS3 | GS4 | GS5 |
| ‘Control’ | 92.70 | 7.30 | 0.00 | 0.00 | 0.00 | 0.00 |
| ‘Wet Feed – Farm’ | 94.00 | 6.00 | 0.00 | 0.00 | 0.00 | 0.00 |
| 'Water – Hatchery’ | 95.30 | 4.70 | 0.00 | 0.00 | 0.00 | 0.00 |
| ‘Feed & Water – Hatchery’ | 92.00 | 7.30 | 0.70 | 0.00 | 0.00 | 0.00 |
| ‘In-House Hatching’ | 92.70 | 6.70 | 0.70 | 0.00 | 0.00 | 0.00 |
|  | Day 29 |  |  |  |  |  |
|  | GS0 | GS1 | GS2 | GS3 | GS4 | GS5 |
| ‘Control’ | 18.70 | 34.70 | 31.30 | 15.30 | 0.00 | 0.00 |
| ‘Wet Feed – Farm’ | 21.20 | 32.50 | 37.70 | 7.90 | 0.70 | 0.00 |
| 'Water – Hatchery’ | 19.30 | 34.70 | 34.70 | 9.30 | 1.30 | 0.70 |
| ‘Feed & Water – Hatchery’ | 13.30 | 34.00 | 38.70 | 12.70 | 0.70 | 0.70 |
| ‘In-House Hatching’ | 17.30 | 34.00 | 36.00 | 12.70 | 0.00 | 0.00 |
|  | Day 36 |  |  |  |  |  |
|  | GS0 | GS1 | GS2 | GS3 | GS4 | GS5 |
| ‘Control’ | 4.70 | 30.00 | 57.30 | 8.00 | 0.00 | 0.00 |
| ‘Wet Feed – Farm’ | 4.00 | 32.90 | 54.40 | 8.70 | 0.00 | 0.00 |
| 'Water – Hatchery’ | 7.90 | 21.20 | 60.30 | 9.90 | 0.00 | 0.70 |
| ‘Feed & Water – Hatchery’ | 2.00 | 28.00 | 59.30 | 10.00 | 0.70 | 0.00 |
| ‘In-House Hatching’ | 4.70 | 26.00 | 62.00 | 7.30 | 0.00 | 0.00 |
